# A conventional PKC critical for both the light-dependent and the light-independent regulation of the actin cytoskeleton in *Drosophila* photoreceptors

**DOI:** 10.1016/j.jbc.2023.104822

**Published:** 2023-05-16

**Authors:** Bih-Hwa Shieh, Wesley Sun, Darwin Ferng

**Affiliations:** Department of Pharmacology, Center for Molecular Neuroscience and Vanderbilt Vision Research Center, Vanderbilt University, Nashville, Tennesse, USA

**Keywords:** eye-PKC, actin cytoskeleton, *Drosophila*, retinal degeneration, *PLCβ*, PLC21C

## Abstract

*Pkc53E* is the second conventional protein kinase C (PKC) gene expressed in *Drosophila* photoreceptors; it encodes at least six transcripts generating four distinct protein isoforms including Pkc53E-B whose mRNA is preferentially expressed in photoreceptors. By characterizing transgenic lines expressing Pkc53E-B-GFP, we show Pkc53E-B is localized in the cytosol and rhabdomeres of photoreceptors, and the rhabdomeric localization appears dependent on the diurnal rhythm. A loss of function of *pkc53E-B* leads to light-dependent retinal degeneration. Interestingly, the knockdown of *pkc53E* also impacted the actin cytoskeleton of rhabdomeres in a light-independent manner. Here the Actin-GFP reporter is mislocalized and accumulated at the base of the rhabdomere, suggesting that Pkc53E regulates depolymerization of the actin microfilament. We explored the light-dependent regulation of Pkc53E and demonstrated that activation of Pkc53 E can be independent of the phospholipase C PLCβ4/NorpA as degeneration of *norpA*^*P24*^ photoreceptors was enhanced by a reduced Pkc53E activity. We further show that the activation of Pkc53E may involve the activation of Plc21C by Gqα. Taken together, Pkc53E-B appears to exert both constitutive and light-regulated activity to promote the maintenance of photoreceptors possibly by regulating the actin cytoskeleton.

Conventional protein kinase C (cPKC) requires both diacylglycerol (DAG) and Ca^2+^ for its activity (1, 2) and is one of the major regulatory proteins following the activation of phospholipase C (PLC) that includes PLCβ and PLCγ (3). PLCγ is one of the key effectors of the growth factor receptors, and cPKC has been shown critical for processes associated with morphological changes leading to the growth and differentiation of cells (4). In contrast, in PLCβ-mediated signaling events such as the visual signaling that takes place in *Drosophila* photoreceptors, in which rhodopsin couples to the heterotrimeric Gq protein leading to the activation of PLCβ4 (NorpA) (5, 6), the role of cPKC has not been fully understood. cPKC has been linked to the regulation of the actin cytoskeleton (7, 8). Indeed, several PKC substrates have been identified and characterized (8). However, the mechanisms by which cPKC regulates cell morphology or the cytoskeleton remain to be explored.

There are two cPKCs expressed in *Drosophila* photoreceptors, eye-PKC (9) and Pkc53E (10), both of which share more than 70% sequence homology. Eye-PKC is critical for the negative regulation of the visual response and is localized in the rhabdomere (11), the visual organelle in which the visual signaling takes place. Moreover, eye-PKC is constitutively associated with a multimeric signaling complex organized by the scaffolding protein INAD (inactivation-no-afterpotential D) (12, 13). Eye-PKC has been shown to phosphorylate both INAD and TRP (transient receptor potential) *in vitro* and *in vivo* (14, 15), both of which are integral parts of the signaling complex. In contrast, the role of Pkc53E in photoreceptors has not been investigated.

Studies have linked cPKCs to the regulation of the actin cytoskeleton (8). The actin cytoskeleton consists of a network of actin microfilaments, which can be found in the cell cortex, the stress fiber, and various extensions including filopodia, lamellipodia, and microvilli. The actin cytoskeleton is critical for maintaining cell shape and it also regulates diverse processes including cytokinesis, chemotaxis, and endocytosis (16).

Here we performed molecular characterization of the *pkc53E* locus and demonstrate that the B-isoform is expressed only in photoreceptors. We show that GFP-tagged Pkc53E-B is present in the cytoplasm and the rhabdomere. We characterized a loss of function allele of *pkc53E* that lacks the transcript for the B isoform and show the mutant undergoes light-dependent retinal degeneration. Further investigations using Actin-GFP reporter demonstrate that a reduction in the *pkc53E* activity leads to defects in the actin cytoskeleton of rhabdomeres. To address the light-dependent regulation of Pkc53E, we first examined the contribution of PLCβ4 (NorpA) that mediates the visual response (17). Unexpectedly, the knockdown of *pkc53E* greatly exacerbated the retinal degeneration of *norpA* mutants, indicating a NorpA-independent regulation of Pkc53E. To explore the alternate pathways leading to the activation of Pkc53E, we show that Plc21C (18) may be involved. Moreover, activation of Plc21C may require Gqα leading to the generation of DAG thereby activating PKC when PLCβ4 is absent.

## Results

### Molecular characterization of the *pkc53E* locus

The *pkc53E* (CG6622) gene is the second cPKC gene in *Drosophila* (9), which is located on the second chromosome about 25 kb 3′ of the previously characterized eye-PKC gene (*inaC*). While eye-PKC has been shown involved in the regulation of visual signaling (11), the role of Pkc53E in photoreceptors has not been investigated.

**Figure 1:**
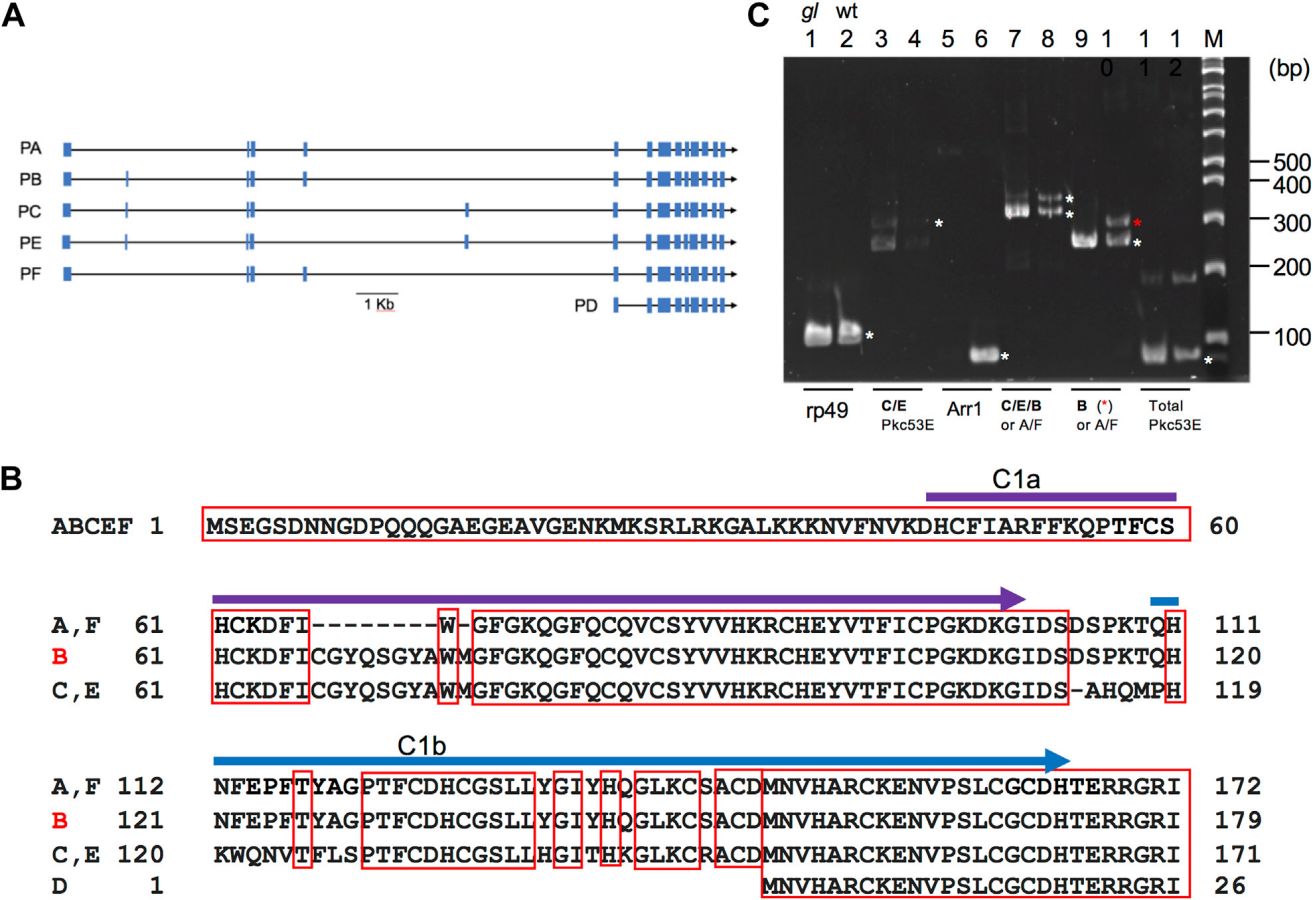
**Molecular characterization reveals that *pkc53-B* is preferentially expressed in photoreceptors.***A*, a graphic map depicting the coding exons (*filled boxes*) of six alternatively spliced transcripts, A to F, in the *pkc53E* locus. *B*, the alignment of the N-terminal sequences from the six Pkc53E isoforms. All isoforms have two distinct C1 domains (*arrows*) of about 50 aa (C1a, aa 45–110, and C1b, aa 120–173, in the B isoform), except the D isoform. Identical amino acids in all isoforms are *boxed in red*. *C*, identification of the photoreceptor-specific isoform by RT/PCR. Shown are PCR products analyzed by polyacrylamide gel (8%). Even-numbered lanes represent products from wild-type and odd-numbered lanes, *glass* mutants (*gl*). Rp49 was served as a positive control whereas arrestin 1 (Arr1), a positive control for wild-type but a negative control for *gl*. DNA fragments corresponding to the predicted PCR products are marked with *asterisks* (∗) next to wild-type lanes. The B isoform of *pkc53E* (*red* ∗) appears highly expressed in photoreceptors as its expression is drastically reduced in the *gl* mutant (lane 9). Specific amplification of genes is indicated below. DNA size standards are shown on the *right*.

Based on the genome annotation at FlyBase, *pkc53E* generates six transcripts (A-F) leading to the translation of four distinct polypeptides with different N-terminal sequences (Fig. 1, *A* and *B*). These Pkc53 E isoforms include PB (679 amino acids, aa), PC/PE (678 aa), PA/PF (670 aa), and PD (525 aa) with variations in the C1 domains that are known to bind DAG (Fig. 1*B*). Specifically, all isoforms contain two C1 domains (C1a and C1b) except PD which lacks 145 aa at the N-terminus. These isoforms are likely to have different affinities toward DAG due to differences in the respective C1 domains (Fig. 1*B*).

We investigated whether any *pkc53E* isoform is expressed in photoreceptors by comparing the expression between wild-type and *glass* (*gl*) mutants that lack photoreceptors (19). We first demonstrated that mRNA for the PB/PC/PE group is present in wild-type (Fig. 1*C*, lane 8) but greatly reduced in heads of *gl* mutants (lane 7), indicating that the PB/PC/PE group is preferentially expressed in photoreceptors. To explore further, we employed isoform-specific primers and observed that the PB isoform was highly expressed in wild-type (Fig. 1*C*, lane 10) but not *gl* heads (lane 9). In contrast, mRNA for the PC/PE group was present in both wild-type and *gl* heads (Fig. 1*C*, lanes 3 and 4). Taken together, our results support the notion that *pkc53E-B* which encodes a polypeptide of 679 aa is preferentially expressed in photoreceptors. Pkc53E-B shares 57% sequence identities with eye-PKC.

### Loss of function in *pkc53E*-B leads to light-dependent retinal degeneration

**Figure 2:**
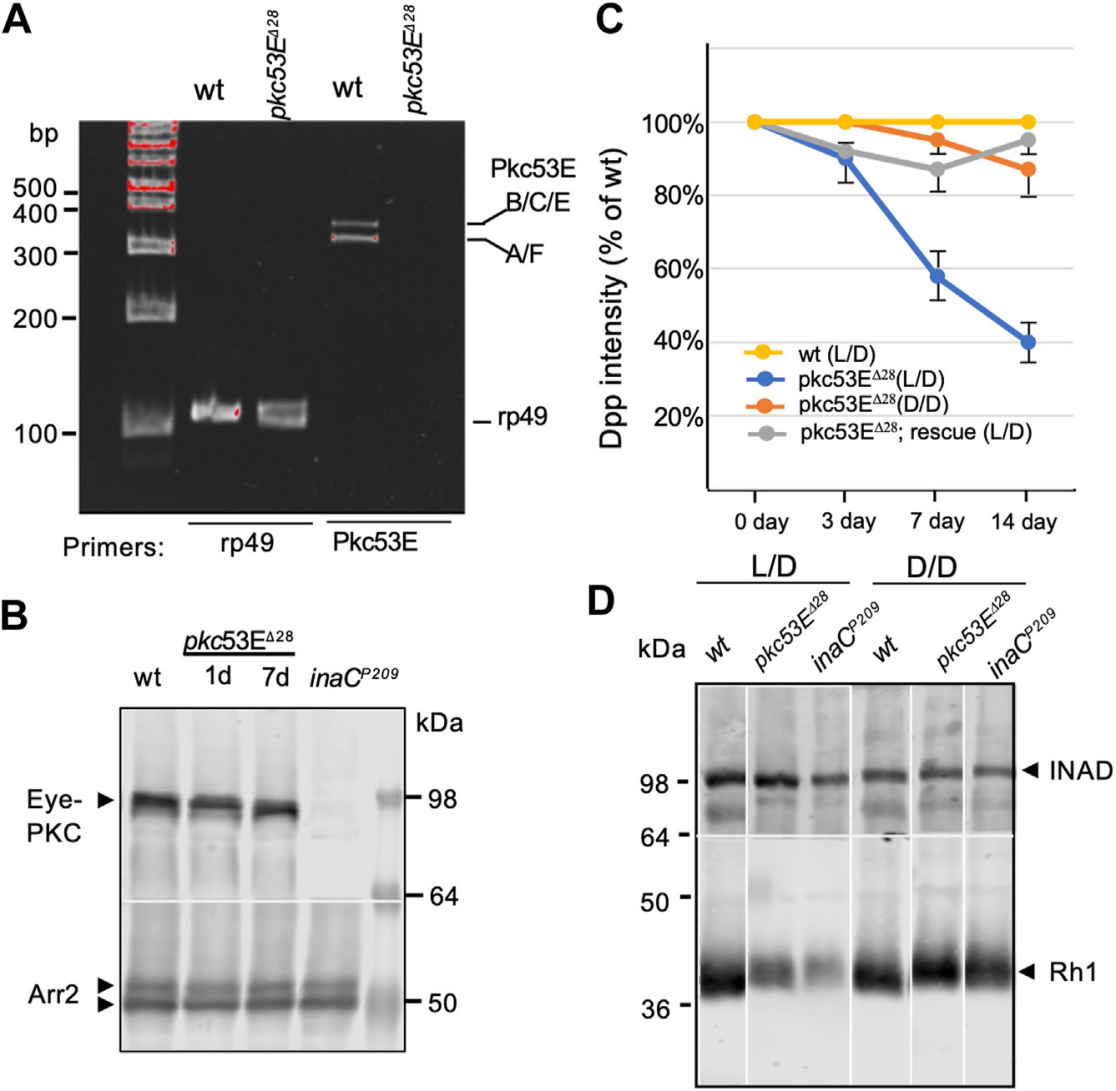
**Loss of function in *pkc53E* leads to light-dependent retinal degeneration.***A*, a greatly reduced expression of *pkc53E* in the null allele, *pkc53E*^*Δ28*^. Shown are the RT/PCR results comparing the expression between the wild-type and the mutant. The *pkc53E* transcripts including A to C, E, and F isoforms are drastically reduced in the mutant (6.2 ± 1.7%, n = 3). DNA size markers are indicated on the *left*. *B*, *pkc53E*^*Δ28*^ does not affect the expression of eye-PKC. Shown is a Western blot consisting of the merged images of the same blot probing with either anti-eye-PKC or anti-Arr2 (loading control) antibodies. Protein molecular weight standards are indicated on the *right*. *C*, the light-dependent retinal degeneration in *pkc53E*^*Δ28*^ and the rescue by the *pkc53E-B-GFP* transgene. Shown are the time courses depicting the intensity of dpp in *pkc53E*^*Δ28*^ and *pkc53E*^*Δ28*^; rescue, under either 12 h L/D (L/D) or constant-dark (D/D) conditions. *D*, the light-dependent retinal degeneration in *pkc53E*^*Δ28*^ or *inaC*^*P209*^ as determined by a reduction of Rh1. Shown is a Western blot consisting of the merged images from the same blot probing with either anti-Rh1 or anti-INAD (loading control) antibodies in extracts from 7-day-old wild-type, *inaC*^*P209*^, and *pkc53E*^*Δ28*^ raised in either L/D or D/D conditions. Selected lanes with representative results from the same Western blot, as indicated, were chosen and assembled.

We obtained a mutant allele, *pkc53E*^*Δ28*^, which was generated by imprecise excision of the P-element in P{EPgy2}Pkc53E^EY14093^ (FBrf0241761). This P-element is located about 2 kb at the 5′ of all isoforms (Fig. S1) except *pkc53E-D*. We analyzed the genomic DNA from the mutant to uncover the extent of the deletion within the *pkc53E* locus; we show that *pkc53E*^*Δ28*^ lacks the 5′ sequence including the first three exons leading to the deletion of the first 76 aa for Pkc53E-B (Figs 1 and S1). In contrast, the 3′ sequence including the fourth exon and beyond remains intact. Taken together, *pkc53E*^*Δ28*^ is devoid of the promoter and some of the N-terminal coding sequence, which would greatly affect the transcription of all *pkc53E* transcripts except the short form, *pkc53E-D*. Consistently, *pkc53E*^*Δ28*^ displays a drastically reduced level of the major *pkc53E* transcripts including that coding for the B isoform, supporting that *pkc53E*^*Δ28*^ is a loss of function allele of *pkc53E-B* (Fig. 2*A*). To ensure that the excision of the P-element did not affect the expression of the adjacent eye-PKC gene, we analyzed the eye-PKC content by Western blotting and showed it is not affected in *pkc53E*^*Δ28*^ (Fig. 2*B*).

To uncover the function of Pkc53E in photoreceptors, we explored whether *pkc53E*^*Δ28*^ undergoes light-dependent retinal degeneration similar to *inaC*^*P209*^ which lacks eye-PKC. Experimentally, we monitored the reduction of deep pseudopupil (dpp) in the eye, which is commonly employed to detect retinal degeneration. Dpp reflects the optical superposition of rhodopsin epifluorescence in the rhabdomere (20). Indeed, the intensity of dpp in *pkc53E*^*Δ28*^ is progressively reduced under 12 h L/D (L/D) but not significantly in the constant dark (D/D) conditions (Fig. 2*C*). We also analyzed the total Rh1 content in the mutants by Western blotting (Fig. 2*D*); we show that 7-day-old *pkc53E*^*Δ28*^ (L/D) contained a reduced Rh1 content (51.2 ± 3.2%), similar to *inaC*^*P209*^ (56.4 ± 1.5%), further supporting that *pkc53E*^*Δ28*^ undergoes the light-dependent retinal degeneration.

### Rescue of *pkc53E*^*Δ28*^*via* transgenic expression of GFP tagged *pkc53E-B*

To investigate whether the light-dependent degeneration defect in *pkc53E*^*Δ28*^ is caused by the loss of the Pkc53E-B isoform specifically, we performed a rescue experiment by transgenically expressing in R1-6 photoreceptors a modified *pkc53E-B* containing an enhanced GFP tag. We show that the transgene prevented the light-dependent reduction of dpp (Fig. 2*C*) and the Rh1 level (Fig. S2) in *pkc53E*^*Δ28*^ under 12 h L/D conditions. These findings further support that a lack of Pkc53E-B is responsible for the light-dependent retinal degeneration in *pkc53E*^*Δ28*^ mutants.

### Subcellular localization of Pkc53E-B *via* GFP tagged *pkc53E-B*

We investigated the subcellular distribution of Pkc53E-B for insights into localization and functions *in vivo*. It is known that cPKC present in the cytosol becomes tethered to the plasma membrane by associating with DAG following the activation of PLC. Subsequently, activated cPKC may translocate close to its substrates by interacting with protein scaffolds or adaptor proteins such as receptors for activated C Kinase (21). For example, a scaffolding protein INAD anchors eye-PKC to a multi-protein signaling complex in the rhabdomere of photoreceptors (12, 13).

**Figure 3:**
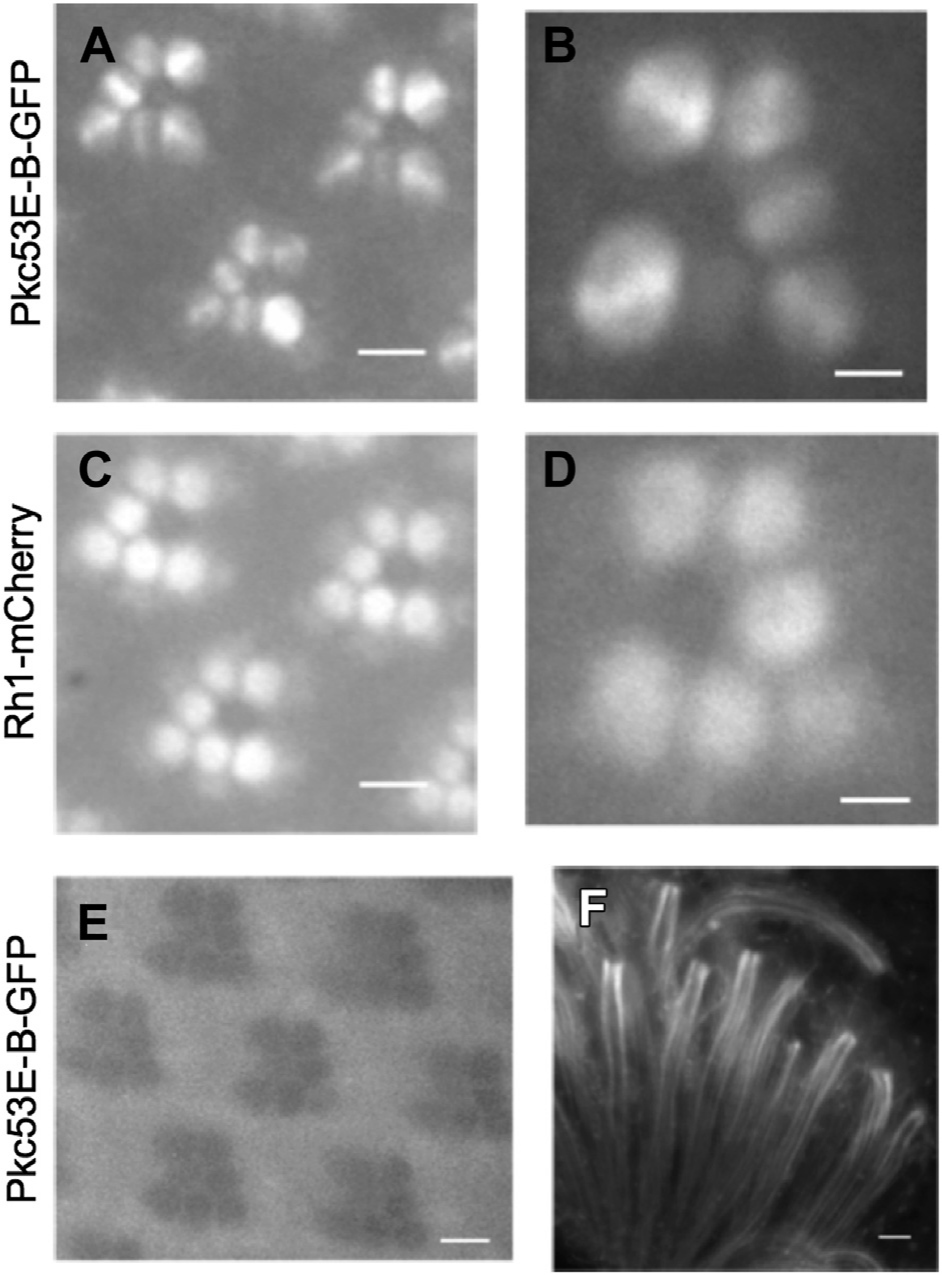
**Subcellular localization of GFP tagged Pkc53E-B in photoreceptors.** The distribution of GFP-tagged Pkc53E-B was analyzed in live retinas (*A* and *B*). Pkc53E-B is detected in the rhabdomere, but also in the cytosol. In the rhabdomere, Pkc53E is not uniformly distributed but appears enriched along the axis of photoreceptors (*B*). In contrast, Rh1-mCherry is uniformly present in the rhabdomere (*C* and *D*). When raised in either constant light or constant-dark conditions, Pkc53E-B is not detected in the rhabdomere but appears distributed and/or sequestered in the cytoplasm (*E*). In the absence of GFP signals, the R1-R7 rhabdomeres appear as clusters of dark circles under the blue light illumination. In dissociated photoreceptors, Pkc53E can be observed in both rhabdomeres and the cytoplasm (*F*). Scale bars, 5 μm (*A*, *C*, and *E*); 2 μm (*B* and *D*); 20 μm (*F*).

To investigate the subcellular distribution of the GFP-tagged Pkc53E-B we employed water-immersion fluorescence microscopy in live retinas. We show that Pkc53E-B could be observed in both rhabdomeres and the cytosol of photoreceptors (Fig. 3). When Pkc53-B was detected in the cytosol, additional blue light stimulation (1300 lux) for 10 min failed to traffic the kinase to the rhabdomere, indicating that membrane recruitment appears not solely regulated by DAG and Ca^2+^ following light stimulation. Furthermore, the distribution and intensity of Pkc53E-GFP in the rhabdomere are not significantly altered when shifting flies to the dark for 1 h (not shown), suggesting that Pkc53E-B is not readily released from the rhabdomere membrane when the visual signaling has been terminated. In contrast, the intensity of the rhabdomere localized Pkc53E-B was reduced upon continued blue light stimulation for 5 min (not shown), suggesting that persistent light stimulation promotes the translocation out of rhabdomeres possibly due to desensitization or downregulation of the kinase. Together, the rhabdomere localization of the kinase is not acutely regulated by DAG but possibly by binding to adaptor proteins. Interestingly, Pkc53E-B is not uniformly distributed but concentrated along the horizontal axis of rhabdomeres (Fig. 3, *A* and *B*), which is consistent with its association with adaptor proteins in the rhabdomere. This unique localization is different from that of Rh1-mCherry which appears uniformly distributed (Fig. 3, *C* and *D*).

We further explored the role of light that influences subcellular localization. Importantly, Pkc53E-B was found only in the cytosol when flies were raised in either constant dark or light conditions (Fig. 3*E*), suggesting that alternating light and dark conditions similar to the diurnal cycle are required for the rhabdomere localization. For example, the diurnal cycle may influence the abundance of the Pkc53E interacting proteins in the rhabdomere. Thus, we propose that adaptor proteins are integral for the localization of Pkc53E-B in rhabdomeres. The identity of the Pkc53E-B interacting proteins and their regulation of the kinase remain to be explored.

### Characterization of retinal degeneration in *pkc53E* mutants and knockdowns in either *eye-PKC* or *pkc53E*

We examined the light-dependent degeneration of the *pkc53E* knockdown caused by RNA-mediated interference (RNAi) (22, 23) and compared it to that of the null mutant and *eye-PKC* knockdown. Specifically, we employed the GMR driver (24) to direct the expression of double-strand RNA using the UAS/GAL4 binary system (25). Subsequently, we examined the retinas of live flies using Arrestin 2-GFP (Arr2-GFP) (26) or Actin-GFP (27, 28). This use of two GFP reporters allows us to explore specifically whether Rh1 or the actin cytoskeleton of the rhabdomere may be affected (Fig. S3).

**Figure 4:**
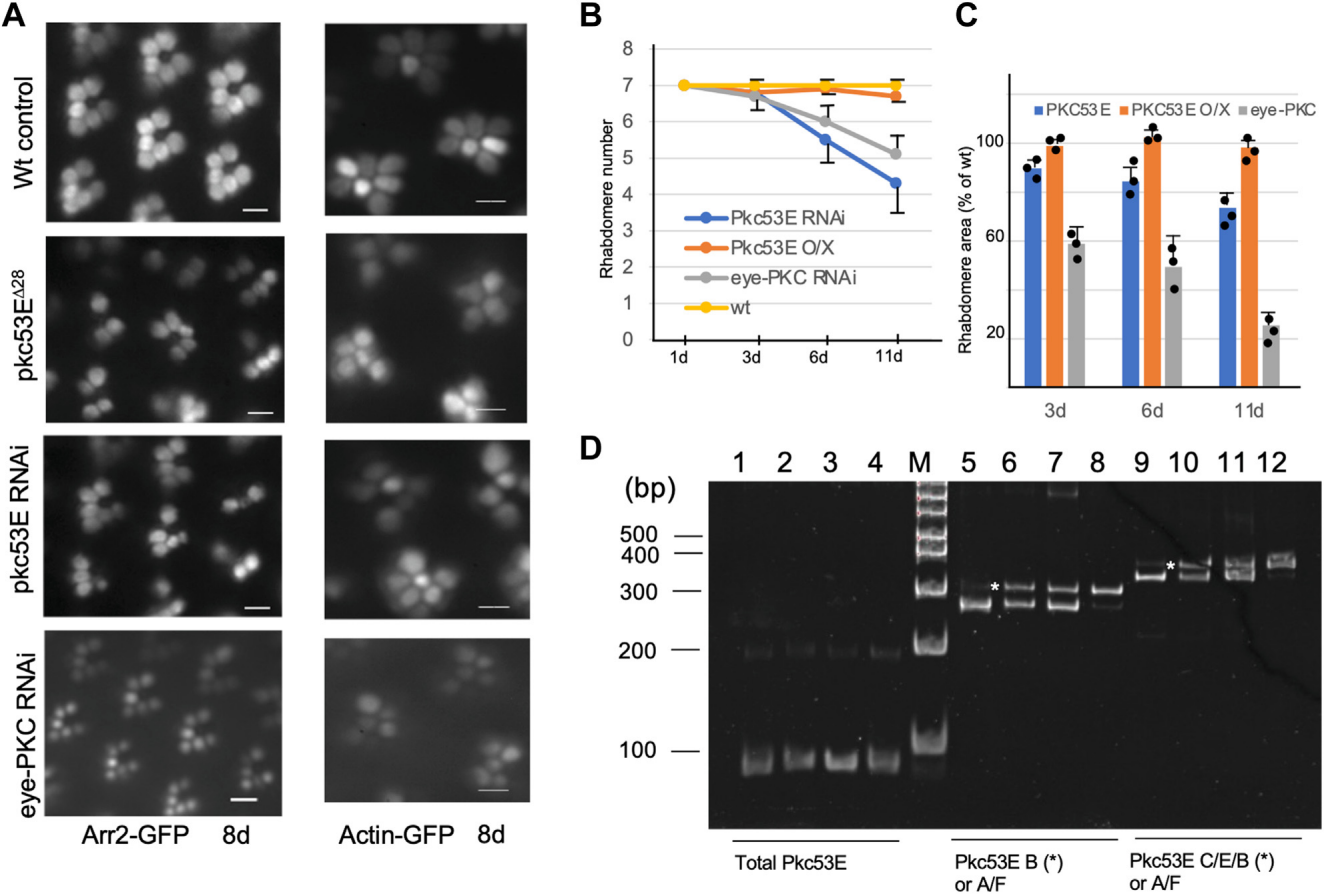
**Knockdown of *pkc53E* or eye-PKC leads to distinct degeneration phenotype.***A*, retinal degeneration caused by *pkc53E*^*Δ28*^ or knockdown of either *pkc53E* or *eye-PKC* using Arr2-GFP (*left*) or Actin-GFP (*right*) as the reporter. Shown are representative retinal morphology of 8-day-old flies. Scale bars on the *left panel*, 5 μm, and on the *right panel*, 2 μm. *B*, comparison of the age-dependent loss of rhabdomeres in various *pkc* mutants (*pkc53E* RNAi, *pkc53E* overexpression, *eye-PKC* RNAi) using Actin-GFP as the reporter. Each time point represents the mean of three flies (mean ± S.E.M, n = 3). *C*, the age-dependent changes of rhabdomere area in *pkc* mutants. Shown are mean ± S.E.M (n = 3) from three independent experiments. *D*, changes of the *pkc53E-B* mRNA expression following photoreceptor-targeted RNAi or overexpression by RT/PCR. Three sets of oligonucleotide primers (below) were used. Shown are PCR products analyzed using 8% polyacrylamide gel. The mRNA level for *pkc53E-B* (∗) is drastically reduced to 5.1 ± 4.1 (n = 3) when compared to the wild-type control of 45.3 ± 8.5 (n = 3, lanes 5). In contrast, overexpression of *pkc53E-B* leads to an increase of about two-fold (203.5 ± 17.2%; lanes 8). The first-strand cDNA templates derived from *pkc53E* RNAi (lanes 1, 5, and 9), wild-type (2, 6, and 10), flies expressing GMR-GAL4 driver alone (3, 7, and 11), and *pkc53E* overexpressing flies (4, 8, and 12) were used. The PCR products corresponding to or enriched with the B-isoform are marked with *asterisks* next to the wild-type lanes.

Using Arr2-GFP we show *pkc53 E*^*Δ28*^ displays an age-dependent retinal degeneration that is characterized by distorted ommatidia clusters with missing rhabdomeres (Fig. 4*A*, left panel), which were also observed in the *pkc53E* knockdown. For example, we detected the loss of about one or two rhabdomeres with 4.8 ± 0.7 (n = 3) remaining in most ommatidia clusters in 8-day-old flies. In contrast, the knockdown of *eye-PKC* led to more severe degeneration with reduced GFP intensity but less impact on the number, orientation, and arrangement of rhabdomeres within the cluster (Fig. 4*A*). The difference in retinal morphology between *eye-PKC* RNAi and *pkc53E* RNAi may reflect distinct underlying mechanisms leading to degeneration.

Conventional PKC has been implicated in regulating the actin cytoskeleton. Therefore, we examined how the actin cytoskeleton of rhabdomeres might be affected using Actin-GFP. We show that both *pkc53E*^*Δ28*^ and *pkc53E* RNAi resulted in reduced or loss of actin cytoskeleton, which is accompanied by the abnormal arrangement of ommatidia clusters (Fig. 4*A*, right). Moreover, in *pkc53E* RNAi, the number of rhabdomeres was reduced from seven to about five in 10-day-old retinas (Fig. 4*B*), a reduction similarly observed using Arr2-GFP. In contrast, *eye-PKC* RNAi initially affected the rhabdomere diameter, and later the rhabdomere number (Fig. 4, *B* and *C*). The age-dependent reduction of the rhabdomere number (Fig. 4*B*) or the area (Fig. 4*C*) in either *pkc53E* or *eye-PKC* knockdown was compared. We also investigated the functional consequence of overexpressing *pkc53E-B* in the wild-type background. However, overexpression did not have a significant impact on the retinal morphology (Fig. 4, *B* and *C*).

Knockdown and overexpression of *pkc53E* were confirmed by RT/PCR, which shows transcripts corresponding to *pkc53E-B* were greatly reduced to about 11.3% of wild-type [from 45.3 ± 8.5 (n = 3) to 5.1 ± 4.1 (n = 3)] (Lanes 5, 9, Fig. 4*D*). In contrast, overexpression increased the mRNA about two-fold (203.5 ±17.2%, n = 3) (Lanes 8, 12, Fig. 4*D*). The knockdown of *eye-PKC* was analyzed by Western blotting that showed the eye-PKC level was decreased to 4.9 ± 3.4% (n = 4) when compared to wild-type (not shown).

Taken together, our findings support the notion that a reduced Pkc53E-B activity mostly affects the integrity of the actin cytoskeleton. We propose that Pkc53E-B is required for the maintenance of rhabdomeres.

### Abnormal distribution of Actin-GFP in *pkc53E* RNAi photoreceptors when raised in the dark

**Figure 5:**
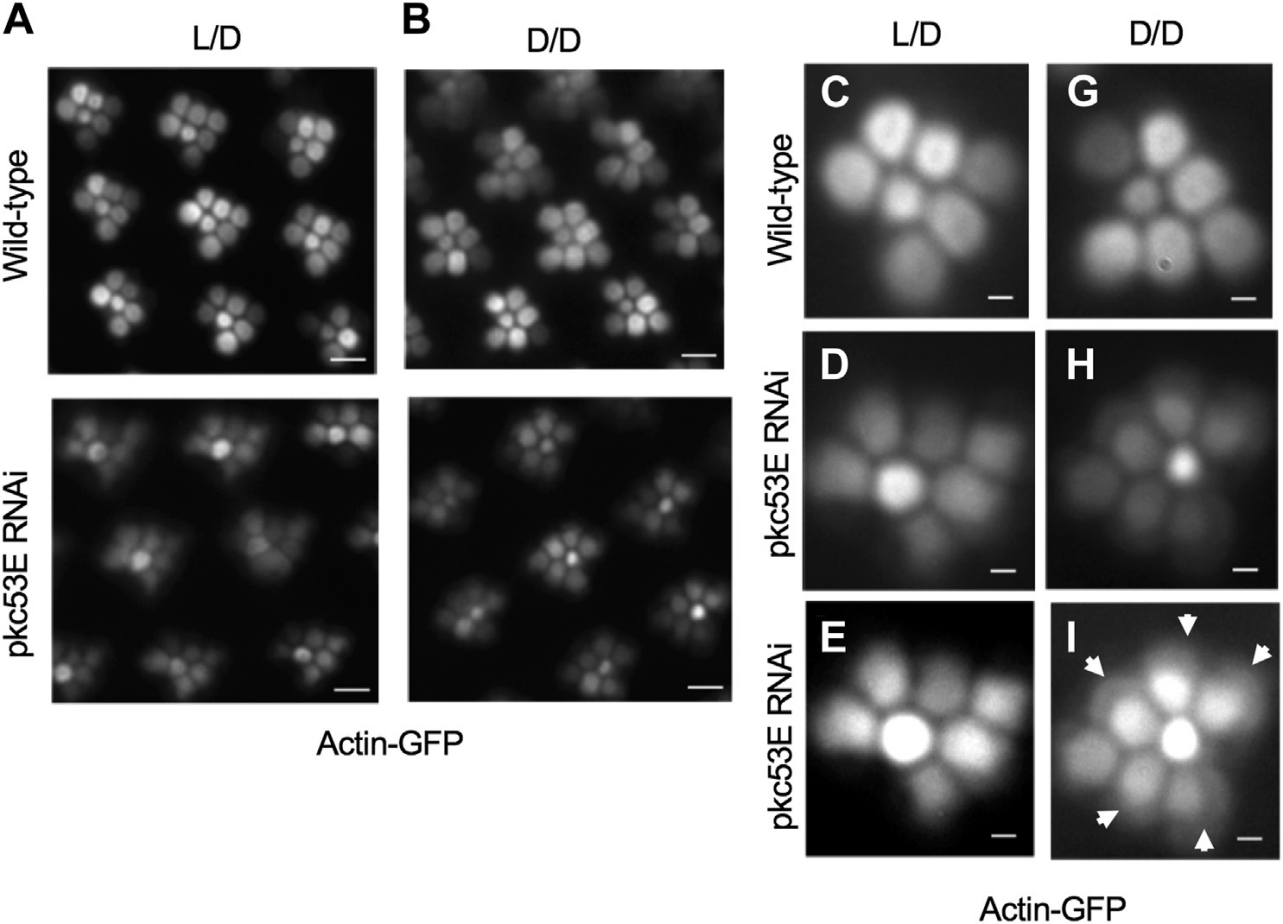
**The light-independent regulation of the actin cytoskeleton by Pkc53E.** A reduced Pkc53E activity impacts the actin cytoskeleton of the rhabdomere in flies raised in either 12 h L/D (*A*) or constant dark (*B*) conditions. Shown are the retinal morphology of wild-type and knockdown mutant marked with Actin-GFP that is enriched in the actin cytoskeleton of rhabdomeres. Scale bars, 5 μm. When examining closely at individual rhabdomere level, Actin-GFP was also present in the cytosol from *pkc53E* knockdown flies raised in D/D (*H*), when compared with those in L/D (*D*). Wild-type controls are shown in (*C*) (L/D) and (*G*) (D/D). Shown below (*E* and *I*) are enhanced images marked with *arrows* to indicate the cytosolic accumulation of Actin-GFP. Scale bars in (*C*–*I*), 2 μm.

To modulate visual sensitivity, many invertebrate photoreceptors exhibit light-dependent turnover of the visual organelle such as rhabdomere (29). We investigated how Pkc53E is involved in the light-dependent remodeling of the actin cytoskeleton in the rhabdomere. We compared the distribution of Actin-GFP in photoreceptors of *pkc53E* knockdown to that of wild-type, both of which were subjected to either constant dark or 12 h L/D conditions for 5 days (Fig. 5). Significantly, we show that in either condition *pkc53E*-RNAi retinas contained smaller rhabdomeres (Fig. 5, *A* and *B*, bottom panel; Fig. 5, *C*, *D*, *G*, and *H*) indicating that Pkc53E is involved in both the light-dependent and light-independent regulation of the actin cytoskeleton. Furthermore, Actin-GFP appeared to accumulate in the cytoplasm including the base of the rhabdomere when *pkc53E*-RNAi flies were raised in constant dark condition (Fig. 5*I*, arrows), compared to those in 12 h L/D (Fig. 5*E*).

Based on the findings, we speculate that Pkc53E exerts at least two different functions in photoreceptors. Particularly, Pkc53E appears critical for the light-independent remodeling of the actin cytoskeleton to ensure the maintenance of the rhabdomere.

### Knockdown of *pkc53E* or *eye-PKC* enhanced the degeneration of *norpA*^*P24*^ photoreceptors

**Figure 6:**
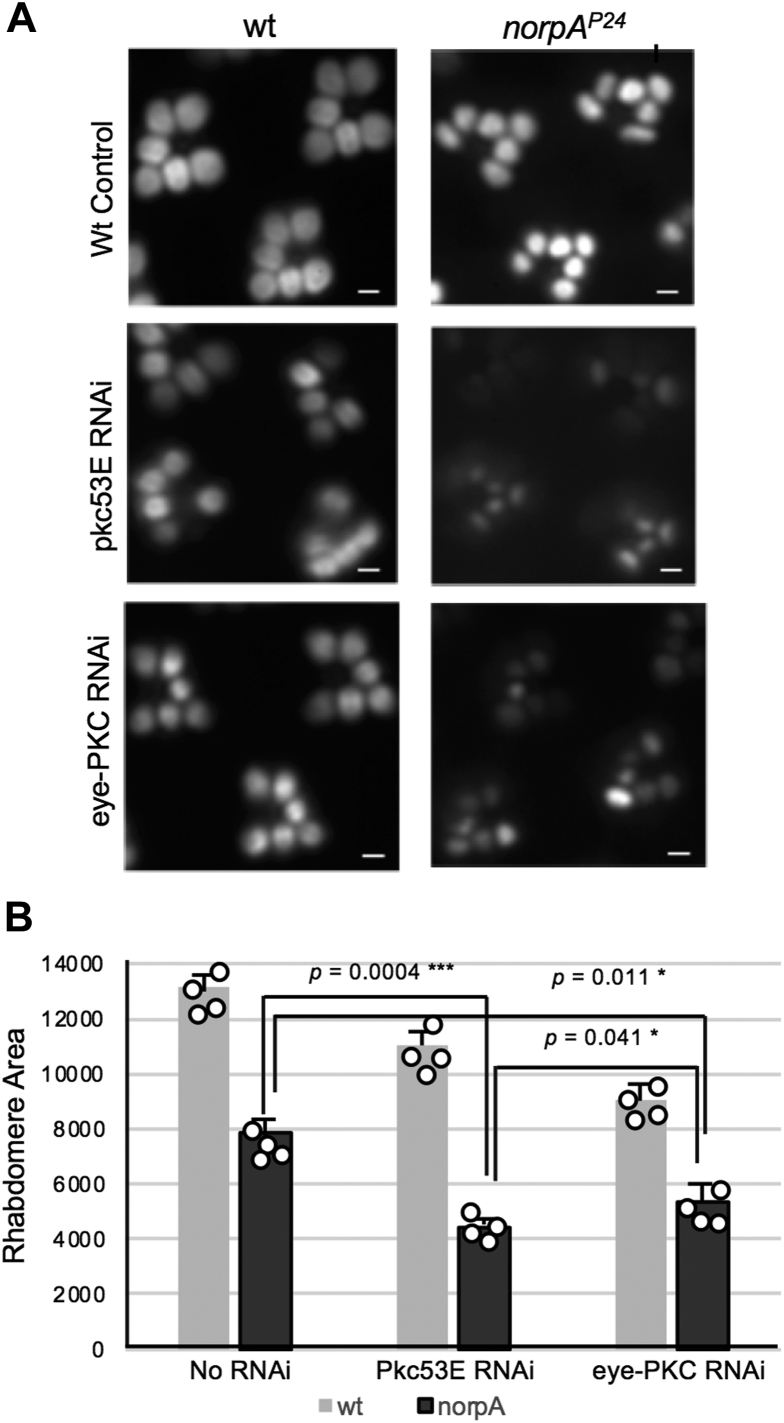
**Genetic interactions between *norpA***^***P24***^**and *pkc53E* or *eye-PKC*.***A*, knockdown of either *pkc53E* or *eye-PKC* enhanced the degeneration of *norpA*^*P24*^ photoreceptors. Shown is the retinal morphology of 7-day-old flies in either wild-type (*left*) or *norpA*^*P24*^ (*right*) genetic background. *norpA*^*P24*^ displays light-dependent retinal degeneration leading to reduced rhabdomeres (*top right*). Similarly, *pkc53E* RNAi (*middle*) and *eye-PKC* RNAi (*bottom*) also result in degeneration. The reduction of rhabdomere size in *norpA*^*P24*^ is further decreased when the activity of either Pkc53E (*middle right*) or eye-PKC (*bottom right*) is decreased. Scale bars, 2 μm. *B*, comparison of rhabdomere areas in wild-type, single and double mutants using Arr2-GFP as the reporter. Shown is a histogram depicting rhabdomere areas (in arbitrary units, n = 4) that show enhancement of degeneration by knockdown of either *pkc53E* or *eye-PKC*. Significant differences between *norpA*^*P24*^ and *norpA*^*P24*^; *pkc53E* RNAi (*p* = 0.0004) or *norpA*^*P24*^ and *norpA*^*P24*^; *eye-PKC* RNAi (*p* = 0.011) were determined by a two-tailed Student’s *t* test.

We explored the light-dependent regulation of Pkc53E and investigated whether Pkc53E is operating downstream of PLCβ4/NorpA that mediates the visual response. It is important to note that *norpA* flies lacking PLCβ4 undergo light-dependent retinal degeneration (26, 30), which is characterized by the age-dependent reduction in rhabdomeres (Fig. 6*A*), a phenotype different from that of *pkc53E* RNAi (Figs. 4 and 6*A*).

We generated and characterized double mutants between *norpA*^*P24*^ and *pkc53E* RNAi. We speculate that double mutants (*norpA*^*P24*^; *pkc53E* RNAi) would lead to a phenotype similar to that of *norpA*^*P24*^ if Pkc53E is acting downstream of PLCβ4 solely. Unexpectedly, we observed more severely degenerated retinas in the double mutant when compared to *norpA*^*P24*^ or *pkc53E* RNAi, indicating that knockdown of *pkc53E* enhances the retinal degeneration of *norpA*^*P24*^ or vice versa (Fig. 6, *A* and *B*). Similarly, the knockdown of *eye-PKC* also accelerated the degeneration of *norpA*^*P24*^ photoreceptors (Fig. 6, *A* and *B*).

Our findings suggested that every single mutant utilizes distinct pathways, which may act synergistically leading to a more severe phenotype in the double mutant. Indeed, it has been proposed that the degeneration of norpA is due to enhanced metarhodopsin-mediated internalization (31) while perturbation of the actin cytoskeleton is likely involved in the degeneration of *pkc53E* RNAi (Fig. 4). Thus, both Pkc53E and NorpA appear to work independent of each other. We conclude that Pkc53E remains active in *norpA*^*P24*^ mutants missing PLCβ4, suggesting the contribution of either another PLCβ or an alternate pathway leading to the synthesis of DAG, such as the activation of phospholipase D (PLD) (26, 30) to activate Pkc53E.

### *plc21C* RNAi but not *pld* RNAi enhanced the degeneration of *norpA*^*P24*^ photoreceptors

**Figure 7:**
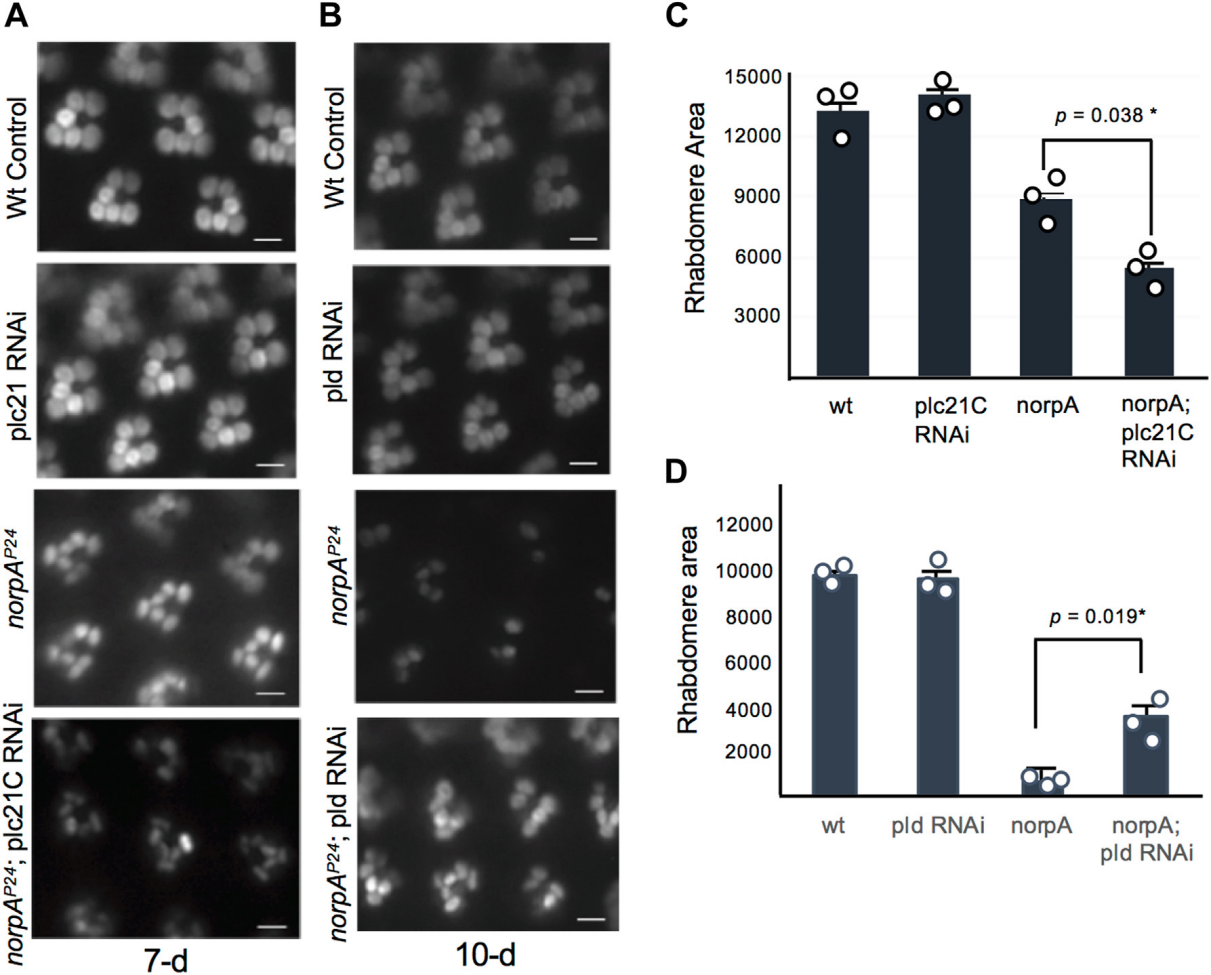
**Knockdown of *plc21 C* enhances the degeneration of *norpA***^***P24***^**photoreceptors.***A*, genetic interactions between *norpA*^*P24*^ and *plc21C* RNAi. *plc21C* RNAi does not affect the retinal morphology in the wild-type background but exacerbates the retinal degeneration of *norpA*^*P24*^ (*bottom panel*). Shown are the retinas of 7-day-old flies using Arr2-GFP as the reporter. Scale bars, 5 μm. *B,* genetic interactions between *norpA*^*P24*^ and *pld* RNAi. *pld* RNAi alone does not modify retinal morphology but delays the degeneration of *norpA*^*P24*^ photoreceptors (*bottom*). Shown are the retinas of 10-day-old flies. Scale bars, 5 μm. *C*, comparison of rhabdomere areas in a histogram to show the enhancement of *norpA*^*P24*^ degeneration by *plc21C* RNAi (in arbitrary units, n = 3). *D,* comparison of rhabdomere areas in a histogram to support the delay of *norpA*^*P24*^ degeneration by *pld* RNAi (in arbitrary units, n = 3). Significant differences between *norpA*^*P24*^ and *norpA*^*P24*^; *plc21C* RNAi (*p* = 0.038) or *norpA*^*P24*^ and *norpA*^*P24*^; *pld* RNAi (*p* = 0.019) were determined by a two-tailed Student’s *t* test.

We first investigated the contribution of phospholipase C at 21C (Plc21C) (18), which was reported to participate in the light-dependent regulation of the circadian clock (32). Plc21C also functions in olfaction (33). We speculate if Plc21C is critical for promoting the activation of Pkc53E, the knockdown of *plc21C* would similarly enhance the degeneration of *norpA*^*P24*^ photoreceptors. Indeed, this was observed as shown in 7-day-old flies (Fig. 7, *A* and *C*). However, *plc21C* RNAi alone did not significantly modify the retinal morphology (Fig. 7, *A* and *C*), indicating that Plc21C is not required or exerts minimal contribution in the wild-type genetic background.

We also investigated whether PLD played a role to activate two cPKCs. PLD is known to catalyze the hydrolysis of phosphatidylcholine to release phosphatidic acid (34), which can be dephosphorylated by Laza to generate DAG (35, 36). It was reported that overexpression of *pld* led to retinal degeneration but rescued degeneration of *norpA* photoreceptors (37, 38). In contrast, a loss of PLD did not affect retinal morphology under 12 h L/D condition (37). We speculate if PLD is critical for promoting the activation of Pkc53E in the *norpA*^*P24*^ background, degeneration of *norpA*^*P24*^ photoreceptors would be enhanced by the knockdown of *pld*.

We characterized 10-day-old *pld* RNAi; *norpA*^*P24*^ and observed the double mutant displayed a better retinal morphology than that of *norpA*^*P24*^ alone, strongly suggesting that activation of PLD is not critical for the Pkc53E activity. Indeed, the knockdown of *pld* appeared to alleviate the degeneration of *norpA*^*P24*^ photoreceptors (Fig. 7, *B* and *D*), suggesting that the PLD activity may lessen the metarhodopsin-mediated retinal degeneration. However, the knockdown of *pld* alone did not significantly modify retinal morphology (Fig. 7, *B* and *D*).

We validated each knockdown by RT/qPCR analyses. We show mRNA levels for *pld* and *plc21C* in the fly head were reduced by 55 ± 14% (n = 3), and 41 ± 11% (n = 3), respectively, following RNAi. As both *pld* and *plc21* are widely expressed in the fly head, the residual mRNA levels may be contributed by the expression in non-photoreceptors.

Taken together, our findings strongly suggest that Plc21C but not PLD is critical for the generation of DAG, thereby activating Pkc53E and eye-PKC in *norpA*^*P24*^ photoreceptors when PLCβ4 is absent.

### *Gqα* RNAi leads to retinal degeneration that is not modified by *pkc53E* RNAi

We explored the mechanism by which Plc21C becomes activated in *norpA*^*P24*^ photoreceptors. Similar to PLCβ4, Plc21C may be activated by the heterotrimeric Gq protein (33, 39). Indeed, Gqα, the α-subunit of Gq, was shown to couple to Plc21C in *norpA*^*P24*^ (32). We speculate if Gqα is directly involved in activating Plc21C leading to the activation of Pkc53E, the knockdown of Pkc53E would not modify the degeneration in *Gqα* RNAi.

**Figure 8:**
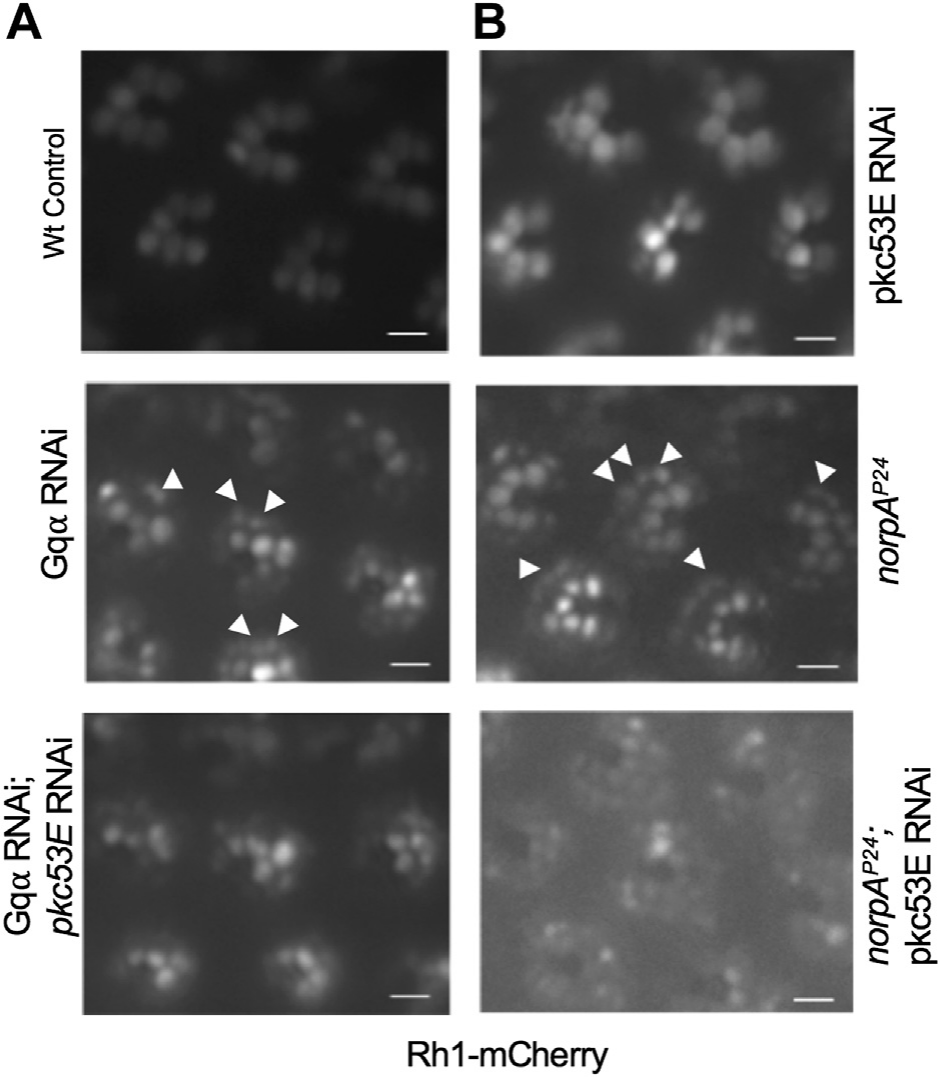
**Genetic interactions between *pkc53E* and *Gqα* using Rh1-mCherry as the reporter.***A*, knockdown of *pkc53E* does not modify retinal degeneration caused by *Gqα* RNAi. In wild-type photoreceptors, Rh1-mCherry is mostly localized in the rhabdomere (*top*). *Gqα* RNAi leads to retinal degeneration that is accompanied by the accumulation of internalized Rh1 rhodopsin (*arrows*, *middle panel*). Retinal degeneration of *Gqα* RNAi is not affected by the knockdown of *pkc53E* (bottom). *B*, knockdown of *pkc53E* enhances the degeneration in *norpA*^*P24*^ photoreceptors. Retinal degeneration in *norpA*^*P24*^ also results in the accumulation of internalized Rh1-mCherry (*arrows*, *middle panel*) similar to that of *Gqα* RNAi. In contrast, *pkc53E* RNAi does not affect the subcellular distribution of Rh1-mCherry (*top*) but enhances the retinal degeneration of *norpA*^*P24*^ (*bottom panel*). Shown are the retinas of 6-day-old flies. Scale bars, 5 μm.

To uncover the relationship between *Gqα* and *pkc53E*, we employed a transgene expressing a modified Rh1 with a mCherry tag (40). In the wild-type background, Rh1-mCherry was mostly localized in the rhabdomere of R1-6 photoreceptors (Fig. 8*A*, top). In contrast, the knockdown of *Gqα* led to retinal degeneration, which is characterized by a reduction of Rh1-mCherry in the rhabdomere and the accumulation in vesicles within the cytoplasm of photoreceptors, as shown in 5-day-old retinas (Fig. 8*A*, middle). This phenotype with internalized Rh1-mCherry was similarly observed in *norpA*^*P24*^ photoreceptors (Fig. 8*B*, middle), consistent with the notion that a reduction of either PLCβ4/NorpA or Gqα activity that greatly diminishes the Ca^2+^ influx appears to impact the recycling of the activated rhodopsin (31, 41).

Importantly, we show that double knockdown (*Gqα* RNAi; *pkc53E* RNAi) and *Gqα* knockdown resulted in a similar phenotype (Fig. 8*A*, bottom), indicating that knockdown of *pkc53E* did not modify retinal degeneration of *Gqα* knockdown. This finding supports that Gqα may be required for the activation of Pkc53E in *norpA*^*P24*^ photoreceptors. Similarly, *eye-PKC* RNAi did not enhance the retinal degeneration caused by *Gqα* RNAi (not shown). Therefore, activation of both eye-PKC and Pkc53E in *norpA*^*P24*^ appears to involve Gqα. Taken together, we propose that Gqα may couple to Plc21C leading to the activation of Pkc53E in *norpA* photoreceptors. Knockdown of the Gq subunit was confirmed by Western blotting using polyclonal antibodies (data not shown).

## Discussion

### Pkc53E-B participates in distinct regulatory mechanisms in photoreceptors

In *Drosophila* photoreceptors, light initiates a cascade of biochemical events leading to the breakdown of PIP_2_ by PLCβ to generate IP_3_ and DAG, which subsequently activates eye-PKC and probably Pkc53E. While eye-PKC plays a major role in the negative regulation of the visual response, the function of Pkc53E remains elusive. Here we report the identification and molecular characterization of a photoreceptor-specific isoform, Pkc53E-B, and show a loss of function of *pkc53E-B* leads to the light-dependent retinal degeneration and light-independent disruption of the rhabdomere actin cytoskeleton. The morphological characteristics of degeneration revealed by two GFP reporters in *pkc53E* mutants are different from those of *inaC* missing eye-PKC, supporting the notion that Pkc53E-B exerts a distinct role different from that of eye-PKC in photoreceptors.

### Pkc53E-B is critical for the light-dependent and light-independent maintenance of the actin cytoskeleton

We explored the contribution of Pkc53E-B in regulating the actin cytoskeleton of photoreceptors. The actin cytoskeleton provides mechanical support for the rhabdomere membrane. It is important to note that the actin cytoskeleton is dynamic as it undergoes constant remodeling by depolymerization at the pointed end and polymerization at the barbed end (42). We show that a reduced or loss of *pkc53E*-B activity negatively impacted the actin cytoskeleton of rhabdomeres independent of the light condition. Furthermore, we observed the accumulation of Actin-GFP in the cytosol including at the base of rhabdomeres, a compartment that is critically involved in membrane cytoskeletal reorganization (43, 44). This phenotype becomes more pronounced in flies that were raised in the constant dark condition, suggesting that Pkc53E-B is involved in the light-independent actin depolymerization.

Pkc53E-B also appears to participate in the light-dependent maintenance of the actin cytoskeleton of rhabdomeres and a loss of Pkc53E leads to retinal degeneration. Pkc53E-B may coordinate the remodeling of the actin cytoskeleton with the light-stimulated turnover of the rhabdomere membrane proteins possibly by regulating proteins that serve as a link between the membrane and the cytoskeleton (44). Mis-regulation of the membrane-cytoskeleton interaction is likely to enhance the turnover of rhabdomere proteins leading to a reduced Rh1 content. As mentioned before, Pkc53E appears to modulate the light-independent remodeling of the actin cytoskeleton by fine-tuning the microfilament depolymerization. Therefore, in the absence of light stimulation, actin becomes accumulated at the base of rhabdomeres of *pkc53E* mutants. However, accumulation of Actin-GFP is not observed in the mutant under 12 h L/D condition, which could be due to the contribution of additional mechanisms and/or the potential involvement of eye-PKC. How does Pkc53E regulate the turnover of actin filaments? We speculate that Pkc53E may regulate actin-binding proteins to perturb the dynamics of actin polymerization and depolymerization (42).

### Subcellular distribution and trafficking of Pkc53E-B in photoreceptors

It is well established that cPKC is recruited to the membrane by binding to DAG following PLC activation. In photoreceptors, Pkc53E-B can be found in both cytosol and rhabdomeres. Translocation between these two compartments appears not acutely affected by the activation of visual signaling as it occurs with much slower kinetics when compared to the light-dependent trafficking of two visual arrestins. For example, Arr2 is rapidly translocated to the membrane with a time constant in seconds upon binding to photoactivated rhodopsin (26, 45). Similarly, translocation of arrestin 1 (Arr1) occurs within minutes of light stimulation (46) upon binding to activated rhodopsin which is also phosphorylated by rhodopsin kinase.

The slow kinetics of rhabdomere translocation by Pkc53E-B strongly suggest that the abundance and/or competence of the Pkc53E-B adaptor/substrate proteins may be subjected to complex regulation. The identity of the Pkc53E-B interacting proteins remains to be investigated. We propose that rhabdomere localized kinase is required for the light-dependent maintenance of photoreceptors critical for averting retinal degeneration. In contrast, cytosolic Pkc53E-B may play a role in the light-independent maintenance of the actin cytoskeleton. Thus, the subcellular localization of Pkc53E-B in photoreceptors supports both the light-dependent and the light-independent activities during the diurnal cycle.

### An alternate pathway involved in the activation of two cPKCs when PLCβ4 is missing

We explored the light-dependent regulation of Pkc53E and observed a reduced Pkc53E activity accelerates retinal degeneration of *norpA*^*P24*^ mutants that lack PLCβ4, the major PLCβ involved in the visual signaling. To explore further how Pkc53E can be activated in *norpA*^*P24*^, we show that the knockdown of Plc21C also exacerbated the degeneration of *norpA*^*P24*^, suggesting that Plc21C may be substituted for PLCβ4 for activating Pkc53E. Our findings support the notion that both PLCβ4 and Plc21C are critical for activating Pkc53E (and eye-PKC). Moreover, Plc21C is required when PLCβ4 is absent.

To explore the upstream regulator of Plc21C, we investigated the role of Gq. We show knockdown of *Gqα* results in retinal degeneration leading to the accumulation of internalized Rh1-mCherry, similar to *norpA*^*P24*^ mutants. Importantly, retinal degeneration of *Gqα* RNAi is not modified by *pkc53E* RNAi, strongly suggesting that Gq is required for the Pkc53E activation. Taken together, we propose that activation of Pkc53E may involve Gqα to turn on Plc21C in *norpA*^*P24*^. It has been shown that a loss of PLCβ4 leads to unregulated Gqα, as NorpA appears to serve as GTPase activating protein for inactivating Gqα (47). Consequently, the unregulated Gq activity may promote its coupling to Plc21C. Together, our results support the notion that the light-dependent activation of Pkc53E involves either PLCβ4 and/or Plc21C, both of which are activated by Gq. In contrast, the light-independent activity of Pkc53E to modulate actin turnover might be due to the basal activity of the kinase.

In visual signaling, activation of rhodopsin leads to an increase of cytosolic Ca^2+^ and transient activation of cPKC. Both Ca^2+^ and cPKC are critically involved in the stability and maintenance of the actin cytoskeleton. Indeed, when the canonical pathway to turn on cPKC is missing, an alternate pathway that utilizes Plc21C is deployed. We propose that regulation of the actin cytoskeleton is a fundamental event that fine-tunes the PLCβ-mediated signaling response.

## Experimental procedures

### Fluorescence microscopy of live photoreceptors

Adult flies were anesthetized by CO_2_ and immobilized in clay with compound eyes facing upward in a 50 mm Petri dish for imaging. The compound eye was examined using an upright Olympus AX70 microscope equipped with a 40X (LUMPlan 40X) or 100X (LUMPlan 100X) water immersion lens for revealing multiple ommatidia of live flies. Image acquisition was performed at 400X or 1000X for rhabdomeres/ommatidia using IPLab image acquisition software (BioVision Technologies) and the Retiga camera from QImaging. Exposure time was made constant throughout each experiment based on the brightest signal in the control group. Multiple flies (n ≥ 3) of each genotype were analyzed. For investigating the subcellular distribution of pkc53E-B-GFP, immobilized flies of the white-eyed genetic background were dark-adapted for 30 min before imaging.

### Fly handling for microscopy

Newly eclosed adult flies were collected and aged under 12 h light/12 h dark (12 h L/D; L, 600 lux) condition unless noted otherwise. For imaging, flies were sorted and manipulated under a dissecting microscope with a light source of 600 lux for less than 1 min. During imaging, compound eyes were subjected to the blue light (1300 lux) or green light (4500 lux) from the fluorescent microscope, and images were taken immediately for Actin-GFP, Arr2-GFP, Pkc53E-B-GFP or Rh1-mCherry.

### Fluorescent image analysis

All image manipulation was performed under the guideline of Rossner and Yamada (48). Fluorescent images included in the Figures are similar in appearance to the raw images. Experimentally, we collected newly eclosed flies of the desirable genotype, placed them in a vial, and aged them at 25 °C for various amounts of time. Live flies were analyzed for GFP or mCherry-marked rhabdomeres by water-immersion fluorescence microscopy. Retinal morphology was scored based on either the rhabdomere area or the number of rhabdomeres present in an ommatidium (unit eye) similar to that described by Cerny *et al.* (49). Briefly, retinas from three flies from each compound eye eight ommatidia were selected and counted to obtain the average of the rhabdomere number. To score rhabdomere areas. Four ommatidia clusters were selected in which the total rhabdomere area of each cluster was measured and averaged *via* ImageJ. Wild-type flies of the same age were used as controls. To score retinal degeneration, we compared the intensity of dpp (20) that is quantified by Image J.

### Quantitative Western blotting

A single fly head was dissected and total proteins were extracted with 15 μl of 2X Laemmli sample buffer by sonication. Proteins were size-fractionated by SDS/PAGE (10–12%) and transferred onto a nitrocellulose filter. Filters were incubated with desired primary antibodies followed by the fluorophore-conjugated secondary antibodies (IRDye 680LT Goat anti-Rabbit IgG, or IRDye 800CW Goat anti-mouse IgG, LI-COR). The fluorophore signal was detected by the Odyssey Infrared Imaging System (LI-COR) and analyzed by Image Studio 5.2.

Individual protein content was normalized by using INAD (50) as the loading control. We carried out three to five analyses using one fly head for each analysis. Polyclonal antibodies against the α-subunit of Gq were generated in rabbits using a bacterial fusion protein corresponding to 1 to 200 aa of *Gqα*. The monoclonal antibody for Rh1 (4C5) was obtained from the Developmental Studies Hybridoma Bank (University of Iowa).

### Recombinant DNA and molecular biology

A full-length Rh1 cDNA lacking the 3′ stop codon was generated by PCR with the engineered SacI (5′) and EcoRI (3′) restriction enzyme sites. The mCherry cDNA sequence with the flanking EcoRI (5′) and XhoI (3′) restriction enzyme sites was generated by PCR using pUAST-mCherry [a gift from Dr Amy Kiger (UCSD)] as the template. The mCherry nucleotide sequence was inserted in-frame into the 3′ of the Rh1 cDNA and the resulting Rh1-mCherry chimera DNA was subcloned into YC4 for the expression under the control of the *Drosophila* Rh1 promoter (26). A full-length cDNA of *pkc53E-B* (GH03188) was obtained from *Drosophila* Genomic Resource Center (Indiana University, Bloomington, IN). The nucleotide sequence of an enhanced GFP was inserted at the 3′ of the *pkc53E* cDNA after the removal of the 3′ stop codon by PCR. The recombinant pkc53E-B-GFP cDNA was subcloned intoYC4 and injected into *yw* embryos.

### Reverse transcription-PCR

Total RNA from 20 fly heads was extracted by a modified method of Chirqwin *et al.* (51) and dissolved in 20 μl water. Five μl of total RNA were used for the first-strand cDNA synthesis *via* Superscript III (Invitrogen) primed with random hexamers. Quantitative PCR in triplicate was performed *via* CFX96 Real-Time System (BIO-RAD) using iQTM SYBR Green Supermix (BIO-RAD). All expression values were normalized to RpL32 (rp49). To compare isoform-specific transcripts in various genetic backgrounds, RT/PCR products were analyzed by polyacrylamide gel (8%) and relative band intensity was quantified using Image Lab (Bio-Rad). Most of the primer sequences used were selected from the FlyPrimerBank (52) and listed below: *rp49* (113 nt), AGCATACAGGCCCAAGATCG (5′), TGTTGTCGATACCCTTGGGC (3′); *pkc53E* (for total *pkc53E*, 90 nt), AGACTCGCACCATTAAGGCTT (5′), GGATGCGTCGATCCTTGTCTT (3′); *pkc53E* (for distinguishing between C/E/B isoforms, 356/359 nt and A/F isoforms, 332 nt), CACGTTCTGCTCCCACTGCA (5′), GCTCCGTGTGATCGCATCC (3′); *pkc53E* (for F isoform, 262 nt or B isoform, 289 nt), AGCCCTCAAGAAGAAGAACGT (5′), TCCTGCGTATGTGAATGGCTC (3′); *pkc53E* for C/E isoforms (282 nt) AGCCCTCAAGAAGAAGAACGT (5′), AGGAAGGTGACATTCTGCCA (3′); GTCGGAGAAACTGGGCAAG (5′), GAAACCGCAGAATGATGGTCC (3′); *arr1* (90 nt), CATGAACAGGCGTGATTTTGTAG (5′), TTCTGGCGCACGTACTCATC (3′); *Plc21C* (159 nt), GAGAAGACAGTGACGGTATGC (5′), CAGGAACATAATCGCCGAGC (3′); Pld (132 nt), GATGAGACCCTCGCTTTTCCT (5′), GACTACACTGTTGTTTTCCTCGT(3′).

### *Drosophila* stocks

*Drosophila* lines including mutants for eye-PKC (*inaC*^*P209*^, #42241) and *Pkc53E* (*pkc53E*^*Δ28*^, #80988) were obtained from Bloomington *Drosophila* Stock Center (BDSC) (NIH P40OD018537). Transgenic flies for GMR-GAL4 (stock #1104), RNAi lines for the following genes including *inaC* (#36776), *pkc53E* (#55864, #27491), *Gqα* (#63987), *plc21C* (#31269, #33719), and *pld* (#32839) were also obtained from BDSC. UAS-driven overexpressing lines including *Pkc53E-B* (#80989), and Actin-GFP (#9253) were from BDSC. Fly cultures were maintained in the standard cornmeal medium at 25 °C. Standard crosses were used to introduce suitable genetic backgrounds.

### Statistical analysis

One-way ANOVA and two-tailed Student's *t* test were employed for statistical analysis.

## Data availability

Data are available within the article or its Supplementary materials.

## Supporting information

This article contains supporting information.

## Conflict of interest

The authors declare that they have no conflicts of interest with the contents of this article.
