# Supplementary material for: A conventional PKC critical for both the light-dependent and the light-independent regulation of the actin cytoskeleton in Drosophila photoreceptors
Source: J Biol Chem. 2023 May 16;299(6):104822. doi: 10.1016/j.jbc.2023.104822 (PMC10318518; doi:10.1016/j.jbc.2023.104822)
Supplement: Supporting Figures S1–S3 [file mmc1.pdf]

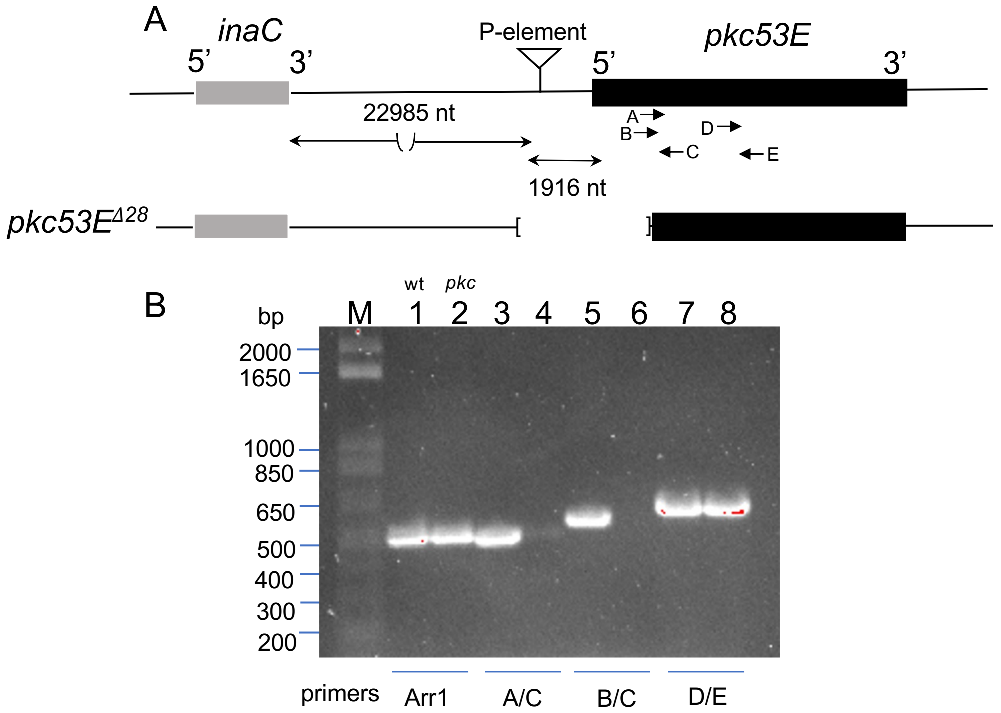

Supplemental Figure 1

Supplemental Figure 1: Mapping of the 5' deletion in the *pkc53E* locus of *pkc53E*$^{\Delta 28}$

A, A diagrammatic representation depicting the genomic region encompassing both *pkc53E* and *eye-PKC* genes. The location of the P-element (EY14093) in the P{EPgy2}Pkc53E$^{[EY14093]}$ line is indicated, which is about 2 kb from the transcription start sites for *pkc53E-B*. The mutant allele, *pkc53E*$^{\Delta 28}$, generated by imprecise excision of the P-element, is missing the 5' sequence including at least 62 amino acids from the initiator methionine, based on genomic PCR analyses. Primer sets used for PCR are listed below and are depicted as arrows in **A**. **B**, Gel electrophoresis of PCR product from mutant and wild-type to support the deletion in the 5' region of *pkc53E*. Shown are PCR products using four sets of primers as indicated below. The Arr1 primers were used for a positive PCR control (508 bp). In contrast, PCR products using 5' primers set A/C (479 bp) and B/C (548 bp) were greatly reduced in the mutant. However, the 3' primer sets (D/E) amplified the coding sequence around 230 aa are present in both the mutant and wild-type to generate a product of 614 bp. Arr1 primer set includes CATGAACAGGCGTGATTTTGTAG (5') and TTCTGGCGCACGTACTCATC (3'); Pkc53E 5' (479 bp), CACGTTCTGCTCCCACTGCA (A), CTACGGCTCGTTCGCCTTGT (C); Pkc53E 5' (548 bp), CACGTTCTGCTCCCACTGCA (B), CTACGGCTCGTTCGCCTTGT (C); Pkc53E 3' (614 bp); AGACTCGCACCATTAAGGCTT (D), ATGGCATACAACTCCTCGCT (E).

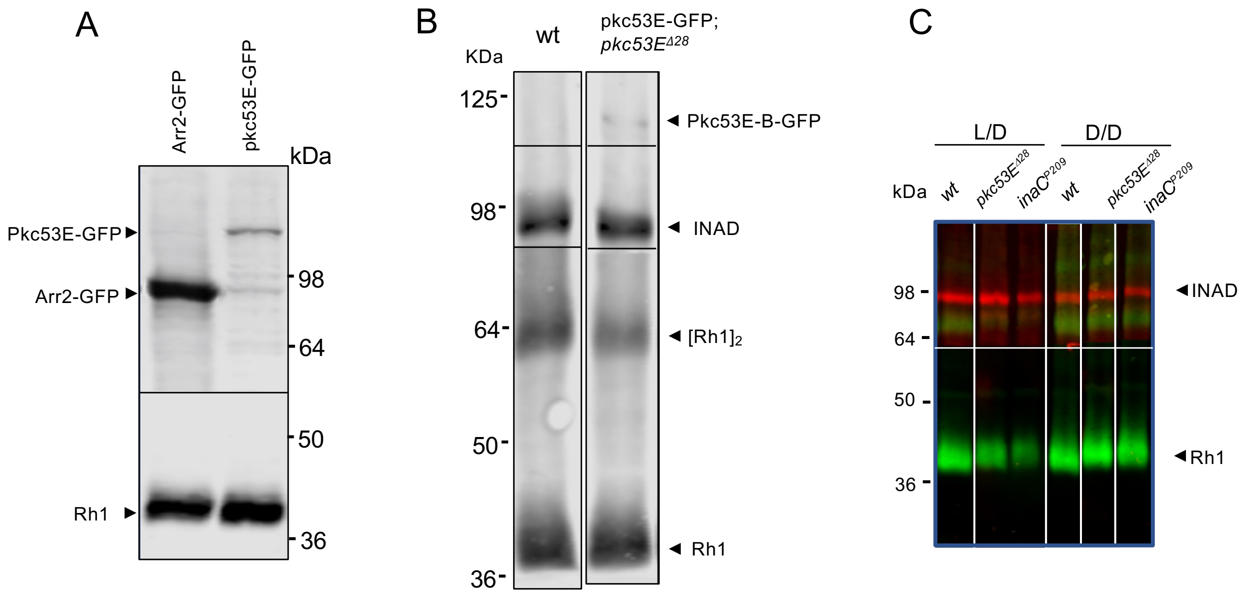

Supplemental Figure 2

Supplemental Figure 2: Rescue of $pkc53E^{\Delta 28}$ by the transgenic expression of *pkc53E-GFP*

A, Transgenic expression of *pkc53E-GFP* by Western blot analysis. Shown is the merged image of the same Western blot probing with anti-GFP antibodies (Invitrogen) or anti-Rh1 monoclonal antibody (4C5). Each lane represents total protein extracts from a single fly head. The Arr2-GFP expressing line was used as a positive control. Protein molecular weight standards are shown on the right. **B**, Rescue of the light-dependent retinal degeneration of $pkc53E^{\Delta 28}$ by the *pkc53E-GFP* transgene. Shown is the result containing the merged images from the same Western blot that measures the Rh1 content, INAD (loading control) or Pkc53E-GFP in 8-day-old flies raised under 12 h L/D condition. $[Rh1]_2$, Rh1 dimers. **C**, The Western blot analysis of $pkc53E^{\Delta 28}$ or $inaC^{P209}$ (7-day-old) as shown in Figure 2D. Shown is the original image of the same Western blot probed with either anti-Rh1 (bottom) or anti-INAD (top) antibodies followed by fluorophore conjugated secondary antibodies. Selected representative lanes from the same blot were chosen and assembled.

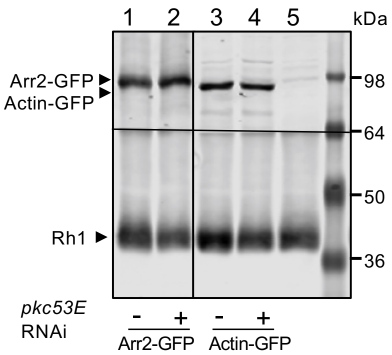

Supplemental Figure 3

Supplemental Figure 3: Levels of Arr2-GFP or Actin-GFP following *pkc53E* RNAi
Shown is a Western blot consisting of the spliced images of the same blot probing with anti-GFP or anti-Rh1 antibody, respectively. Each lane represents a single head of 7-day old flies from control or *pkc53E* knockdown. The level of Rh1 or the respective GFP reporter was determined. Lane 5 contains extracts from wild-type flies that served as the negative control for the GFP transgenes.
